# RETRACTED: Tzeyung et al. Fabrication, Optimization, and Evaluation of Rotigotine-Loaded Chitosan Nanoparticles for Nose-To-Brain Delivery. *Pharmaceutics* 2019, *11*, 26

**DOI:** 10.3390/pharmaceutics16060720

**Published:** 2024-05-27

**Authors:** Angeline Shak. Tzeyung, Shadab Md, Subrat Kumar Bhattamisra, Thiagarajan Madheswaran, Nabil A. Alhakamy, Hibah M. Aldawsari, Ammu K. Radhakrishnan

**Affiliations:** 1 School of Postgraduate Studies, International Medical University, Kuala Lumpur 57000, Malaysia; angelineshak@hotmail.com; 2 Department of Pharmaceutics, Faculty of Pharmacy, King Abdulaziz University, Jeddah 21589, Saudi Arabia; nalhakamy@kau.edu.sa (N.A.A.); haldosari@kau.edu.sa (H.M.A.); 3 Department of Pharmaceutical Technology, School of Pharmacy, International Medical University, Kuala Lumpur 57000, Malaysia; Thiagarajan@imu.edu.my; 4 Department of Life Sciences, School of Pharmacy, International Medical University, Kuala Lumpur 57000, Malaysia; 5 Department of Pathology, School of Medicine, International Medical University, Kuala Lumpur 57000, Malaysia; ammu_radhakrishnan@imu.edu.my

The journal retracts the article, “Fabrication, Optimization, and Evaluation of Rotigotine-Loaded Chitosan Nanoparticles for Nose-To-Brain Delivery” [1]. 

Following publication, concerns were brought to the attention of the publisher regarding an overlap of images.

Adhering to our complaint’s procedure, an investigation was conducted that confirmed a partial duplication between Figures 6A and 6B, presenting different experimental conditions. 

While the authors fully cooperated with the Editorial Office during the investigation, they were unable to satisfactorily explain the overlap of the figures, nor could they meet the required quality standards of raw images in order to consider a correction as per the journal’s original image requirements policy (https://www.mdpi.com/journal/pharmaceutics/instructions#oriimages). As a result, the Editorial Board and Editor-in-Chief were unable to confirm the reliability of the findings and subsequently decided to retract this paper.

This retraction was approved by the Editor-in-Chief of the journal *Pharmaceutics*.

The authors did not agree to this retraction. 
